# Comparison of Multi-Compartment Cable Models of Human Auditory Nerve Fibers

**DOI:** 10.3389/fnins.2019.01173

**Published:** 2019-11-05

**Authors:** Richard Bachmaier, Jörg Encke, Miguel Obando-Leitón, Werner Hemmert, Siwei Bai

**Affiliations:** ^1^Department of Electrical and Computer Engineering, Technical University of Munich, Munich, Germany; ^2^Munich School of Bioengineering, Technical University of Munich, Garching, Germany; ^3^Medizinische Physik and Cluster of Excellence Hearing4all, Universität Oldenburg, Oldenburg, Germany; ^4^Graduate School of Systemic Neurosciences, Ludwig Maximilian University of Munich, Planegg, Germany; ^5^Graduate School of Biomedical Engineering, University of New South Wales, Sydney, NSW, Australia

**Keywords:** auditory nerve, computational model, biophysical, cable model, electrical stimulation, threshold

## Abstract

**Background:** Multi-compartment cable models of auditory nerve fibers have been developed to assist in the improvement of cochlear implants. With the advancement of computational technology and the results obtained from *in vivo* and *in vitro* experiments, these models have evolved to incorporate a considerable degree of morphological and physiological details. They have also been combined with three-dimensional volume conduction models of the cochlea to simulate neural responses to electrical stimulation. However, no specific rules have been provided on choosing the appropriate cable model, and most models adopted in recent studies were chosen without a specific reason or by inheritance.

**Methods:** Three of the most cited biophysical multi-compartment cable models of the human auditory nerve, i.e., Rattay et al. (2001b), Briaire and Frijns (2005), and Smit et al. (2010), were implemented in this study. Several properties of single fibers were compared among the three models, including threshold, conduction velocity, action potential shape, latency, refractory properties, as well as stochastic and temporal behaviors. Experimental results regarding these properties were also included as a reference for comparison.

**Results:** For monophasic single-pulse stimulation, the ratio of anodic vs. cathodic thresholds in all models was within the experimental range despite a much larger ratio in the model by Briaire and Frijns. For biphasic pulse-train stimulation, thresholds as a function of both pulse rate and pulse duration differed between the models, but none matched the experimental observations even coarsely. Similarly, for all other properties including the conduction velocity, action potential shape, and latency, the models presented different outcomes and not all of them fell within the range observed in experiments.

**Conclusions:** While all three models presented similar values in certain single fiber properties to those obtained in experiments, none matched all experimental observations satisfactorily. In particular, the adaptation and temporal integration behaviors were completely missing in all models. Further extensions and analyses are required to explain and simulate realistic auditory nerve fiber responses to electrical stimulation.

## 1. Introduction

Multi-compartment cable models of the auditory nerve fibers (ANF) have been developed to assist in understanding and predicting neural responses to external stimulation. They have been used to advance our knowledge regarding how the auditory nerve encodes timing, frequency and intensity information (Imennov and Rubinstein, 2009). Moreover, multi-compartment ANF models have been combined with three-dimensional volume conduction models of the human cochlea to simulate responses to cochlear implant (CI) stimulation (Rattay et al., 2001a; Kalkman et al., 2015; Malherbe et al., 2016; Nogueira and Ashida, 2018). Alongside psychophysical experiments, computational models of the auditory nerve are used to evaluate new sound coding and stimulation strategies and are therefore crucial for the improvement of CIs. Nevertheless, there exist several ANF models in the literature with varied morphological or ionic channel properties. Choosing the appropriate cable model for a given computational study is difficult as the different models are difficult to compare based on the original publications. Consequently, most models adopted in existing studies were chosen without a specific reason or by inheritance.

Generally speaking, multi-compartment models are morphological extensions of single-node models. Based on the Schwarz–Eikhof (SE) node model of rat and feline ion channel kinetics (Schwarz and Eikhof, 1987), Frijns et al. (1994) developed an axon model, which was subsequently extended with dendrite and soma to match the feline ANF morphology (Frijns et al., 1995). However, differences in morphology between human and cat might impact spike travel time, and this must be taken into account for correct predictions of CI stimulus coding in humans (Rattay et al., 2001b; O'Brien and Rubinstein, 2016). Therefore, this feline ANF model was later modified to account for the human ANF morphology (Briaire and Frijns, 2005). Meanwhile, Rattay et al. (2001b) designed a different human ANF model based on Hodgkin's and Huxley's (HH) description of the unmyelinated squid axon (Hodgkin and Huxley, 1952) while also including human ANF morphology. Smit et al. (2008) adopted the dendrite and soma from Rattay et al. (2001b) but modified the properties of the axon in order to account for differences in membrane currents at the node of Ranvier between human (Schwarz et al., 1995) and squid.

In addition to differences in morphology and ion channel properties, some ANF cable models also include modifications in order to implement specific physiological properties, including stochastic effects and adaptation. For instance, Rattay et al. (2001b) incorporated a simple and efficient approach to predict stochastic ANF responses by adding a Gaussian noise current term to the total ion current. In comparison, Imennov and Rubinstein (2009) and Negm and Bruce (2014) represented the stochastic nature of ion channels by applying a channel-number tracking algorithm. Woo et al. (2010) included a model of rate adaptation based on a dynamic external potassium concentration, whereas van Gendt et al. (2016) integrated their biophysical model with a phenomenological approach to simulate threshold fluctuations, adaptation and accommodation.

Differences in the description of ANF morphology and physiology lead to distinct model characteristics. A meaningful comparison based on the respective publications is however not feasible, as the models were only fitted to specific ANF properties under certain stimulation patterns. For example, Rattay et al. (2001b) detailed the initiation and propagation of action potentials (APs) but did not describe properties like the strength-duration relation and refractory period. Frijns et al. (1994) and Smit et al. (2008) measured the AP shape, conduction velocity, strength-duration relation and refractory period, but none of these properties were mentioned for the updated versions of their model in Briaire and Frijns (2005) and Smit et al. (2010). Studies that included an adaptation mechanism in their ANF cable models investigated almost exclusively responses to pulse-train stimulation, but did not include single-pulse responses as in other studies. Therefore, it is necessary to compare the spiking characteristics of different ANF models in order to investigate how the models behave with more generalized stimuli. In this study, three often-cited biophysical human ANF cable models—the Rattay (RA) model from Rattay et al. (2001b), the Briaire-Frijns (BF) model from Briaire and Frijns (2005), and the Smit-Hanekom (SH) model from Smit et al. (2010)—were chosen and implemented in a consistent framework, and their performance was evaluated by comparing them against experimental data. It should be noted that all chosen models represent type I spiral ganglion neurons.

## 2. Methods

**Figure 1:**
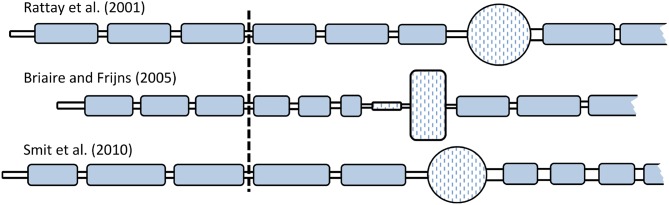
Comparison of the ANF morphologies. All dendrites and axons were myelinated, denoted by the blue color. The somas of all three models were unmyelinated but surrounded by layers of “satellite cells,” as described in Rattay et al. (2001b), and so was the pre-somatic region of the BF model. Relative differences in compartment size among the three models are indicated in the figure, but they are not true to scale. Vertical line indicates the position of the stimulation electrode (distance from the neuron was 500 μm).

The multi-compartment ANF models by Rattay et al. (2001b), Briaire and Frijns (2005), and Smit et al. (2010), from here on abbreviated as RA, BF, and SH, respectively, were implemented in a single framework using Python 3.4, with the package Brian2 (Goodman and Brette, 2009). All models followed the morphology of a human ANF as described in the original publication and consisted of dendrite, soma, and axon. Dendrite and axon were composed of an alternating structure of active nodes and passive myelinated internodes. Additionally, all models included a peripheral terminal as well as a pre-somatic region. All morphological components were modeled as electrical circuits and represented by cylindrical compartments. The spherical shape of the somas in the RA and SH models was approximated by segmenting it into ten cylindrical compartments. Compartment lengths and diameters were distinct in each model, as shown in Figure 1. Details of the morphologies are included in Appendix (Supplementary Material). The length of dendritic internodes in Briaire and Frijns (2005) was defined as scalable so as to reflect the varied lengths from the organ of Corti to the soma. In this study, the dendritic internodes were scaled as suggested by Kalkman et al. (2014) with a maximum length of 250 μm.

In unmyelinated compartments of the ANF models, the cell membrane was represented by a capacitor which was charged or discharged by ionic currents. These currents depended on the membrane's ionic permeabilities and Nernst potentials of individual ion species. All three models included exclusively sodium and potassium channels. The BF model utilized the gating properties suggested by Schwarz and Eikhof (1987) and calculated the ionic currents according to Frankenhaeuser and Huxley (1964), whereas RA and SH adopted the gating properties and equations proposed by Hodgkin and Huxley (1952). However, compared to the original gating properties of the Hodgkin-Huxley (HH) kinetics, which were measured in a squid at 6.3 °C, in the RA and SH models they were each multiplied by a compensating factor to account for the faster gating processes in mammalian nerve fibers, and the ionic channel densities were increased. Furthermore, in order to specifically account for the human ANF physiology, Smit et al. (2010) added two modifications to the HH ion channels in the axon: (a) the opening and closing of the potassium channels were modified to be slower (Smit et al., 2008); (b) a persistent sodium current was added to account for the total sodium current together with a transient one of the original HH model (Smit et al., 2009).

Regarding the passive internodes, Briaire and Frijns (2005) implied that they were surrounded by a perfectly insulating myelin sheath. As a consequence, both their capacity and conductivity were assumed to be zero, whereas Rattay et al. (2001b) described them as a passive resistor-capacitor network and thus as imperfect insulators. In Smit et al. (2010), the dendritic internodes were modeled following Rattay et al. (2001b), but the axonal internodes were described using a double-cable structure as proposed by Blight (1985). Detailed information regarding the ionic models can again be found in Appendix (Supplementary Material).

The extracellular space of the ANF models was simulated as a homogeneous medium with an isotropic resistivity of 3 Ω m. Unless otherwise stated, each fiber was stimulated externally by a point electrode situated above the third dendritic node with a vertical distance of 500 μm to the fiber. Measurements were performed at the tenth axonal node to ensure the propagation of an action potential (AP) to the axon. For each of the properties investigated in this study, the parameters for the applied stimuli were taken from the respective physiological experiments in order to ensure a meaningful comparison with experimental results in the literature. Whenever a biphasic stimulus was administered, it was always cathodic-first.

While the models by Briaire and Frijns (2005) and Smit et al. (2010) in the original studies were deterministic, Rattay et al. (2001b) incorporated a simple approach to predict stochastic ANF responses by adding a Gaussian noise current term to the total ion current. In this study, this simple stochastic approach was added to all models to investigate the stochastic and temporal behaviors (sections 3.6, 3.7). The Gaussian noise current term was calculated with:

(1) $$i_{noise}=X\;\cdot \;k_{\text{noise}}\sqrt{Ag_{\text{Na}}},$$

where *X* is a Gaussian random variable (mean = 0, S.D. = 1). *g*_Na_ denotes the maximum sodium conductivity, and *A* is the membrane surface area. The term is multiplied with the factor *k*_*noise*_, which is common to all compartments and is used to adjust how strongly the stochastic behavior of the channels is emphasized.

## 3. Results

### 3.1. Thresholds

The threshold current *I*_*th*_ of an ANF model is defined as the minimal current amplitude required to elicit an AP with otherwise constant stimulation parameters. This section reports the dependency of *I*_*th*_ on the phase length and polarity of single monophasic pulses, the pulse rate and duration of biphasic pulse trains, and the frequency and duration of sinusoidal stimuli.

#### 3.1.1. Single Monophasic Pulses

**Figure 2:**
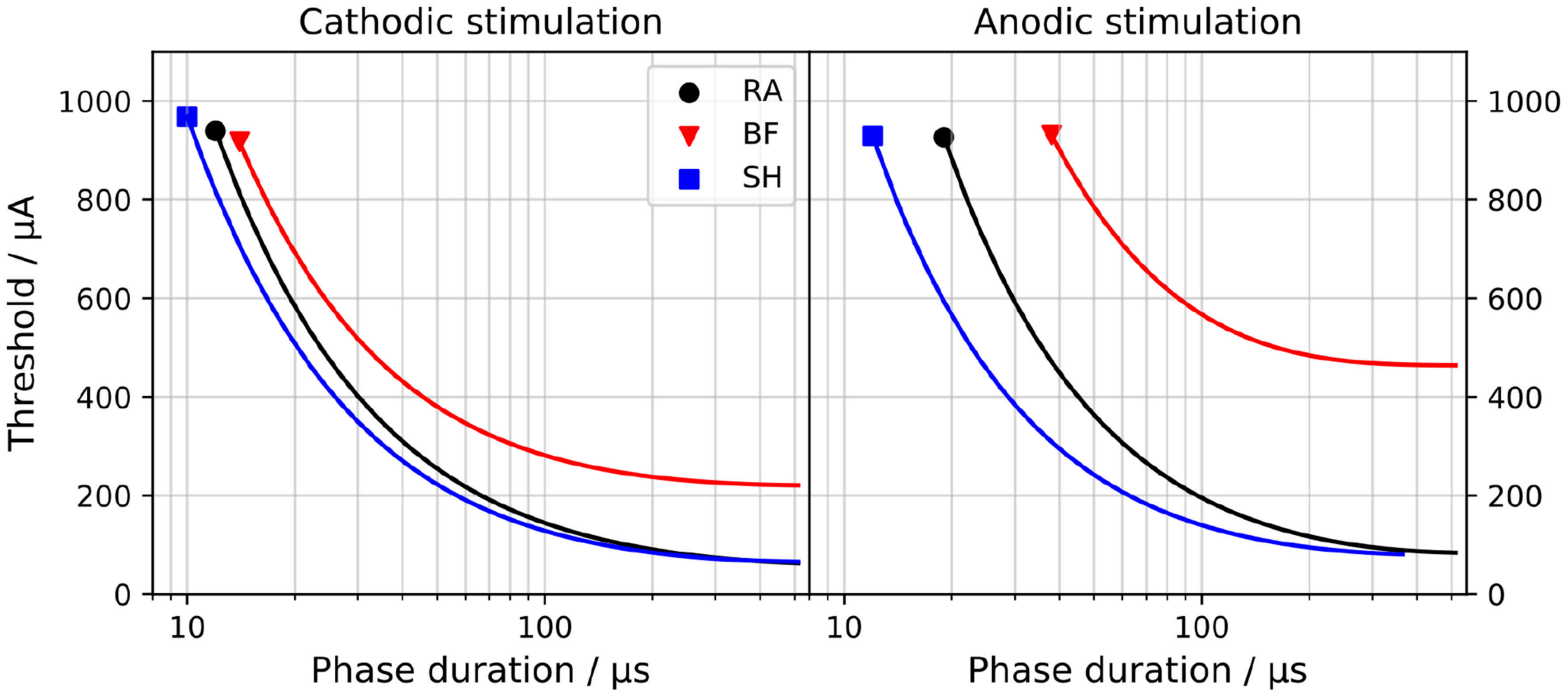
Strength-duration curves for monophasic cathodic **(Left)** and anodic **(Right)** stimuli. “RA,” “BF,” and “SH” denote the Rattay, Briaire-Frijns and Smit-Hanekom models, respectively. The *x*-axis is set in a log-scale for a better comparison.

Figure 2 compares the strength-duration curves, i.e., the relations between *I*_*th*_ and the duration of the applied pulse, for both monophasic cathodic and anodic stimuli. All models demonstrated thresholds that decrease with longer pulse duration. Thresholds were also larger for anodic stimulation; this was most obvious for the BF model.

The current threshold to which a strength-duration curve converges for a very long pulse is called rheobase *I*_*rh*_; the chronaxie τ_*chr*_ defines the required pulse width to elicit an AP when applying twice *I*_*rh*_. These two values are commonly used to characterize the strength-duration behavior of a nerve fiber and are compared among the three models in Table 1. The values for *I*_*rh*_ with cathodic stimuli ranged from 61.3 μA (RA) to 220 μA (BF) and were smaller than those with anodic pulses. While *I*_*rh*_ for the two polarities differed by a factor of 1.4 and 1.2 for the RA and SH model, the threshold for anodic stimulation increased by more than a factor of 2.1 in the BF model. The impact of polarity on τ_*chr*_ was less pronounced, and the values ranged from 39.1 μs (BF) to 125 μs (RA).

**Table 1:** Rheobase *I*_*rh*_ and chronaxie τ_*chr*_ of ANF models for monophasic cathodic and anodic stimulation.

	***I*_*rh*_/μA**		**τ_*chr*_/μs**	
	**Cathodic**	**Anodic**	**Cathodic**	**Anodic**
Rattay model	61.3	83.4	125	122
Briaire-Frijns model	220	464	39.1	39.1
Smit-Hanekom model	64.7	79	93.8	85.9

*The point electrode was situated above the third dendritic node with a vertical distance of 500 μm to the fiber*.

In Ranck (1975), τ_*chr*_ of mammalian nerve fibers were found to lie between 29 and 100 μs, whereas van den Honert and Stypulkowski (1984) suggested a distinctly longer average chronaxie of 264 μs based on experiments with feline ANF. Variations in these experimental observations may be due to differences in experimental setup and stimulation method (Frijns et al., 1994). BeMent and Ranck (1969) measured that anodic pulses required 3.19–7.7 times the current of cathodic pulses to excite feline nerve fibers, and Armstrong et al. (1973) reported a ratio of 1.0–3.2. Therefore, despite the large variation between the three models, all of them show τ_*chr*_ within the experimental range, and all three are consistent with the increased anodic thresholds.

#### 3.1.2. Biphasic Pulse Trains

**Figure 3:**
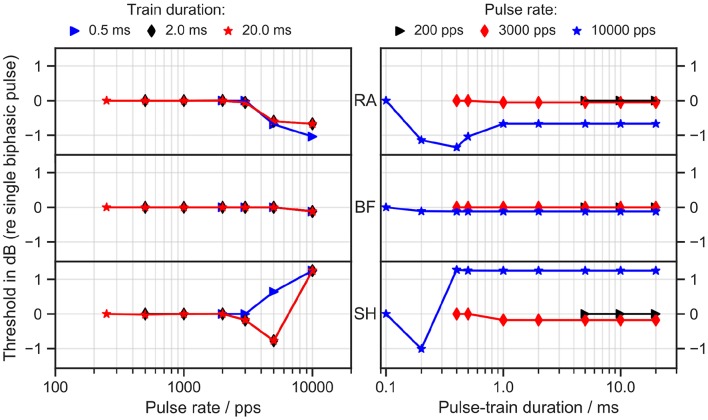
Threshold as a function of pulse rate **(left column)** and pulse-train duration **(right column)**. RA, Rattay model; BF, Briaire-Frijns model; SH, Smit-Hanekom model. The stimulation current was a train of biphasic cathodic-first 45 μs pulses with an inter-phase gap of 8 μs. The threshold is reported in dB as the ratio of *I*_*th*_ for the pulse train to *I*_*th*_ for a single biphasic pulse.

Trains of biphasic pulses with 45 μs/phase and an 8 μs inter-phase gap were applied to all ANF models. *I*_*th*_ was measured as a function of pulse rate and train duration, as depicted in Figure 3. In all cases, the thresholds remained constant for pulse rates up to 2,000 pulses per second (pps) and train durations longer than 1 ms. The RA model predicted a decreasing threshold for pulse rates higher than 2,000 pps with a maximal drop of 1 dB from the single biphasic pulse threshold at 10,000 pps. SH, however, showed an opposite trend: the threshold at 10,000 pps rose by over 1 dB for all train durations longer than 0.3 ms. No obvious differences from the single pulse threshold were observed in BF.

Experiments with human CI listeners have also shown that thresholds decrease with pulse rates (multi-pulse integration). Carlyon et al. (2015) measured a drop of 3.9 dB from 71 to 500 pps and a larger drop of 7.7 dB from 500 to 3500 pps.

Integration for pulse rates even smaller than 10 pps has been observed by Zhou et al. (2015), who delivered pulse-train stimuli through CIs in humans and guinea pigs. They also discovered temporal integration up to 640 ms. Our simulation results thus lead to the conclusion that none of the models were able to predict pulse-train integration in a comparable range with the experimental data.

#### 3.1.3. Sinusoidal Stimulation

**Figure 4:**
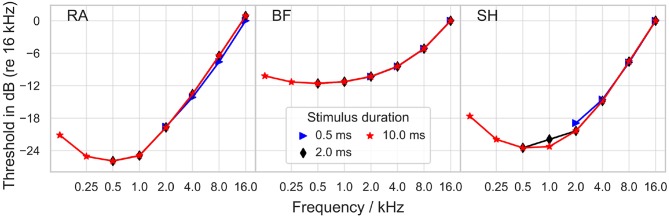
Threshold for sinusoidal stimulation as a function of stimulus frequency. The threshold is reported in dB as the ratio to *I*_*th*_ at the frequency of 16 kHz. “RA,” “BF,” and “SH” denote the Rattay, Briaire-Frijns and Smit-Hanekom models, respectively. All results are plotted for three stimulus durations.

*I*_*th*_ was also measured for sinusoidal stimuli (positive phase first), with frequencies between 125 and 16 kHz, as depicted in Figure 4. All models predicted the minimal threshold at a frequency of 500 Hz. In RA, a growth of approximately 6 dB per octave was obtained for frequencies higher than 1 kHz, and a similar increase, namely 7 dB per octave, was found in SH above 2 kHz; in comparison, BF predicted smaller threshold increases between 1 and 8 kHz; between 8 and 16 kHz the slope was close to 7 dB per octave. Stimulus duration exerted only minimal impact on the threshold.

Dynes and Delgutte (1992) recorded threshold currents in cat auditory nerve fibers. While for high frequencies (8–20 kHz), the slope of the threshold increase approaches 6 dB per octave in most fibers as in the models, for low frequencies (200 Hz–1 kHz) the slope flattened only to about 3 dB per octave and never increased. Shannon (1983) measured the threshold of sinusoidal stimuli with frequencies between 30 Hz and 3 kHz in human CI users. The resulting threshold-frequency curve could be divided into three parts: a rather flat segment for frequencies below 100 Hz, a segment with an increase of 12–15 dB per octave at frequencies between 100 and 300 Hz, and a 3 dB per octave increase segment for higher frequencies. Pfingst (1988) also reported an increase in the threshold of roughly 3 dB per octave for frequencies between 1 and 16 kHz. Pfingst (1988) and Pfingst and Morris (1993) obtained threshold-frequency curves which dropped for small frequencies with a minimum threshold between 60 Hz and 200 Hz. Due to these differences, it must be concluded that the comparison of psychophysical threshold and single fiber recordings/simulations must be taken with a grain of salt.

None of the ANF models predicted a threshold increase of more than 10 dB per octave as measured by Shannon (1983) between 100 and 300 Hz. The threshold-frequency curves predicted with the models dropped between 125 and 500 Hz, so the minimum was reached for a higher frequency than in experiments. The threshold increase measured from BF between 2 and 8 kHz matched the experimental results, whereas the other two models overestimated it by a factor of two.

In the absence of electrophysiological measurements however, psychoacoustic measurements might give an insight into general trends.

### 3.2. Conduction Velocity

The conduction velocity *v*_*c*_ describes how fast an AP propagates along the nerve fiber. Hursh (1939) found in feline nerve fibers that *v*_*c*_ increased linearly with the fiber outer diameter *D*, and reported the scaling factor *k* to be 6. *k* is was defined as

(2) $$k=\frac{v_{c}/\left(ms^{-1}\right)}{D/\text{μm}}.$$

**Figure 5:**
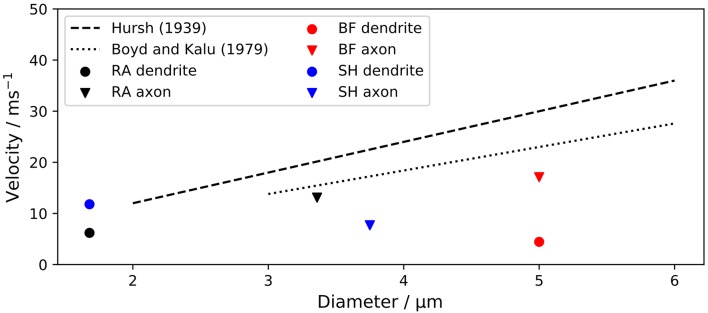
Conduction velocity *v*_*c*_ of ANF models in comparison to experimental data. The velocities of dendrite and axon of each model were measured separately due to their morphological and physiological differences. *v*_*c*_ is plotted against the fiber outer diameters. “RA,” “BF,” and “SH” denote the Rattay, Briaire-Frijns and Smit-Hanekom models, respectively.

Boyd and Kalu (1979) obtained a slightly smaller scaling factor of 4.6 for feline nerve fibers, with an outer diameter between 3 and 12 μm. Figure 5 compares the conduction velocities of ANF models with experimental results.

The velocities of dendrite and axon were measured separately due to their morphological and physiological differences. Scaling factors for the dendrite of BF and the axon of SH were considerably smaller than experimentally obtained values, while all other scaling factors were within ±25 % of the experimental results.

**Figure 6:**
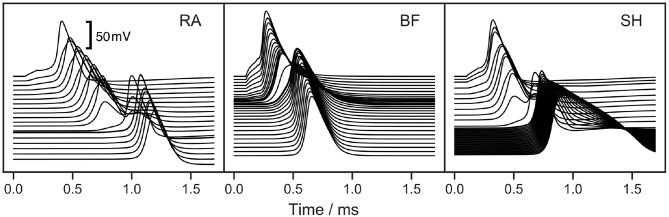
Response of ANF models to a 100 μs cathodic current pulse injected at the peripheral terminal. “RA,” “BF,” and “SH” denote the Rattay, Briaire-Frijns and Smit-Hanekom models, respectively. Each line depicts the voltage over a course of time at a single morphologic component, starting from the peripheral terminal represented by the topmost line. The lines are vertically aligned true to scale according to the compartmental distances. The high capacitance of the soma causes a large additional delay of the AP.

The soma of all three ANF models has a high capacitance due to its large diameter and reduced myelination. Consequently, the soma delays the conduction of APs. This is apparent in Figure 6, which illustrates the model responses to a 100 μs cathodic current pulse injected at the peripheral terminal. The duration of the somatic delay was determined by measuring the time difference between the APs at the nodes directly before and after the soma, which were found to be 305, 130, and 240 μs for RA, BF, and SH, respectively. Stypulkowski and van den Honert (1984) measured the electrically evoked compound AP of feline auditory nerves and observed two peaks with a time difference of 200 μs. They suggested that the earlier peak arose from a direct excitation of the axon near the soma, whereas the second peak had its origin at the dendrite. Accordingly, the time difference between the two peaks can be used to estimate the somatic delay for feline ANFs, which is closer to the values from BF and SH. On the other hand, the double peaks exhibited in neuronal response telemetry measurements with CI listeners have a temporal distance of 300 μs (Lai and Dillier, 2000). Using this value as a reference point for human ANFs, the somatic delay predicted by RA appears very realistic.

### 3.3. Action Potential Shape

**Figure 7:**
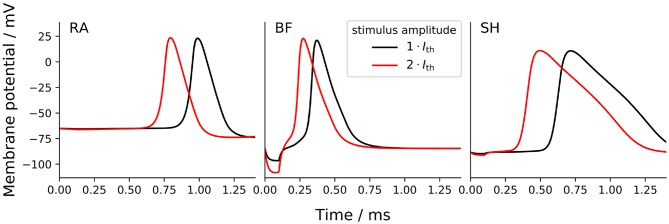
Transmembrane voltage (action potential) at the tenth axonal node of the ANF models to a monophasic 100 μs cathodic current pulse with an amplitude of *I*_*th*_ and 2 × *I*_*th*_. “RA,” “BF,” and “SH” denote the Rattay, Briaire-Frijns and Smit-Hanekom models, respectively.

The shape of AP was compared among ANF models by measuring the height as well as the rise and fall times of AP. The AP height was defined as the voltage difference between the resting potential and the peak value. Rise and fall times were determined as the time periods between the AP maximum and its 10 % height, obtained during the ramp-up and -down phases, respectively. In this section, APs were triggered by a monophasic 100 μs cathodic current pulse with an amplitude of *I*_*th*_ and 2 × *I*_*th*_, as shown in Figure 7.

The increase of the stimulus amplitude by a factor of two resulted in no significant changes in the AP shape in any of the models but drastically shortened their latency, which is reported in section 3.4. The short hyperpolarization at the beginning of the curves from BF was a passive response to the external cathodic stimulus, which is not visible in the other models; this variation may likely be due to the difference in distance between the stimulating electrode (at the third dendritic node) and the recording electrode (at the tenth axonal node) as a result of different internodal lengths among the three models. Another striking feature observed from Figure 7 is the extremely long fall time of 712 μs with SH, which is more than three times as large as those with the other models. In comparison, the differences in AP height and rise time were relatively small: the AP height ranged from about 88 mV (RA) to 107 mV (SH), and all APs peaked at positive values; the rise time ranged from 87 μs (BF) and 121 μs (SH). These parameters that define the AP shape were almost independent of pulse form, phase duration, and stimulus amplitude.

Only a limited number of studies with the objective to investigate AP shape can be found in the literature. Paintal (1966) measured AP rise and fall times of feline nerve fibers at 37.1 °C and revealed an inverse relation with the conduction velocity. The rise time curve was steep for a conduction velocity below 40 m/s and flattened out for faster conduction. On the other hand, the relation between the fall time and conduction velocity was approximately linear. Based on the conduction velocities reported in section 3.2, the data from Paintal (1966) were used to interpolate rise and fall times of the models. The interpolated rise time values for RA, BF, and SH are roughly 220, 190, and 270 μs, respectively, whereas their fall times are longer and range from 350 to 365 μs. As a result, all three ANF models showed distinctly shorter rise times than interpolated values based on Paintal (1966). The fall time values of RA and BF were also smaller than results obtained by Paintal (1966), but the value of SH was about twice as much as the interpolated value. In addition, a recent computational study confirmed the simulated contribution of type I spiral ganglion cells with an AP duration of approximately 1/3 ms, which was close in timing with the experimentally recorded electrically evoked compound action potential (Miller et al., 2004; Rattay and Danner, 2014).

### 3.4. Latency

The latency is defined as the time period between the onset of a stimulus and the peak of the resulting AP. Four monophasic cathodic stimuli differing in phase duration and stimulus amplitude were applied to the ANF models, and the corresponding latency was measured at the third dendritic node, which was right below the electrode. Results are listed in Table 2 along with values from feline experiments. All models predicted a shorter latency than the experimental data for all considered stimuli, with RA in general having the closest values to experimental measurements and BF producing significantly smaller latency values than the other models. This could partly be due to determining the latency at the compartment closest to the electrode in the model while, in the experiment, it might have been determined further away from the spike initiation site which would add an conduction delay. In both experiment and model, increases in phase duration led to a longer latency, while an increase in the amplitude resulted in a shorter latency. Nevertheless, the data from van den Honert and Stypulkowski (1984) suggest a latency reduction of around 50% when doubling the stimulation current (Stim. B to Stim. C). RA and BF predicted a larger decrease of around 69% and 66% while SA predicted 57%.

**Table 2:** Action potential latency of ANF models measured with four different stimuli.

	**Stim. A**	**Stim. B**	**Stim. C**	**Stim. D**
Rattay model	275 μs	283 μs	87 μs	323 μs
Briaire-Frijns model	140 μs	148 μs	50 μs	193 μs
Smit-Hanekom model	261 μs	267 μs	115 μs	298 μs
*Cartee et al. (2000)*	440 μs	–	–	–
*van den Honert and Stypulkowski (1984)*	–	685 μs	352 μs	–
*Miller et al. (1999)*	–	–	–	650 μs

*Latency values from relevant feline studies are also included (italicized)*.

*A: monophasic 40μs cathodic current pulse with amplitude I_th_*.

*B: monophasic 50μs cathodic current pulse with amplitude I_th_*.

*C: monophasic 50μs cathodic current pulse with amplitude 2I_th_*.

*D: monophasic 100μs cathodic current pulse with amplitude I_th_*.

### 3.5. Refractoriness

The refractoriness characterizes the reduced excitability of an ANF after the initiation of an AP. It was measured in this study as described in Frijns et al. (1994): two monophasic 50 μs cathodic stimuli were applied. The first stimulus with an amplitude of 1.5*I*_*th*_ served as a masker for the second one; the current threshold of the second stimulus, necessary to elicit another AP, was measured for different inter-pulse intervals (IPI), i.e., the time period between the two stimuli (Wesselink et al., 1999).

**Figure 8:**
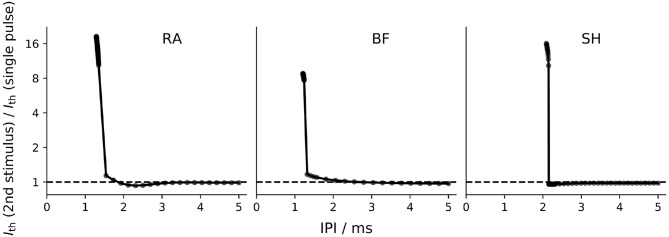
Refractory curve of ANF models. Both the masker and the second stimulus were a monophasic cathodic pulse with a phase length of 50 μs. “RA,” “BF,” and “SH” denote the Rattay, Briaire-Frijns and Smit-Hanekom models, respectively. Please notice that the scaling of the *y*-axis is logarithmic.

Figure 8 depicts the refractoriness of the ANF models. In this figure, the relative increase in threshold of the second stimulus compared to a single pulse threshold is plotted against the IPI. At small IPI values, the refractory curves of all models showed a steep decrease, where the thresholds of the second stimulus quickly approached the masker threshold. For IPI values around 2 ms, RA and SH predicted the threshold of the second pulse slightly smaller than the single pulse threshold.

The refractoriness of an ANF can be described by the absolute and relative refractory periods: the absolute refractory period (ARP) is the period after the initiation of an AP, during which it is impossible for a second propagating AP to be elicited regardless of the strength of stimulus; the subsequent period that requires an elevated threshold for spike generation is called the relative refractory period (RRP). In this study, ARP was recorded as the time interval between two stimuli, during which the second stimulus required a current amplitude of at least 4 times the masker amplitude to elicit a second AP, whereas RRP was the time period between the two stimuli, where the threshold of the second stimulus was only increased by a factor of 1.01 (Wesselink et al., 1999). The ARP and RRP of ANF models for different stimuli are listed in Tables 3, 4 along with values obtained in feline experiments. All models predicted a smaller RRP than the experimental measurements. Regarding ARP, a larger value than experimental observations was found. In particular, the ARP magnitude of the SH model was twice as large as that of the other models. In the case of BF with a biphasic stimulus of 50 μs/phase, secondary activation was elicited in the model, which resulted in difficulty in determining the ARP in this situation. This was not present in all other situations. While the experimentally measured RRP values were approximately ten times larger than ARP, the ANF models predicted a ratio smaller than two.

**Table 3:** Absolute refractory period (ARP) of ANF models measured with four stimuli.

	**Stim. A**	**Stim. B**	**Stim. C**	**Stim. D**
Rattay model	1381 μs	1372 μs	1333 μs	1331 μs
Briaire-Frijns model	1261 μs	1262 μs	1224 μs	?
Smit-Hanekom model	2151 μs	2143 μs	2105 μs	2139 μs
*Miller et al. (2001)*	334 μs	–	–	–
*Stypulkowski and van den Honert (1984)*	–	300 μs	–	–
*Dynes (1996)*	–	–	500 μs to 700 μs	–
*Brown and Abbas (1990)*	–	–	–	500 μs

*Measurements from feline studies are also included (italicized). The question mark represents a difficulty in determining the exact ARP due to the secondary activation caused by biphasic stimuli in the Briaire-Frijns model*.

*A: monophasic 40μs cathodic current pulses*.

*B: monophasic 50μs cathodic current pulses*.

*C: monophasic 100μs cathodic current pulses*.

*D: biphasic 50μs cathodic first current pulses*.

**Table 4:** Relative refractory period of ANF models measured with four stimuli.

	**Stim. A**	**Stim. B**	**Stim. C**
Rattay model	1.82 ms	1.77 ms	1.28 ms
Briaire-Frijns model	2.43 ms	2.55 ms	2.45 ms
Smit-Hanekom model	2.14 ms	2.11 ms	1.89 ms
*Stypulkowski and van den Honert (1984)*	3–4 ms	*-*	*-*
*Cartee et al. (2000)*	4–5 ms	*-*	*-*
*Dynes (1996)*	*-*	5 ms	*-*
*Hartmann et al. (1984)*	*-*	*-*	5 ms

*Measurements from feline studies are also included (italicized)*.

*A: monophasic 50μs cathodic current pulses*.

*B: monophasic 100μs cathodic current pulses*.

*C: biphasic 200μs cathodic first current pulses*.

### 3.6. Stochasticity

**Figure 9:**
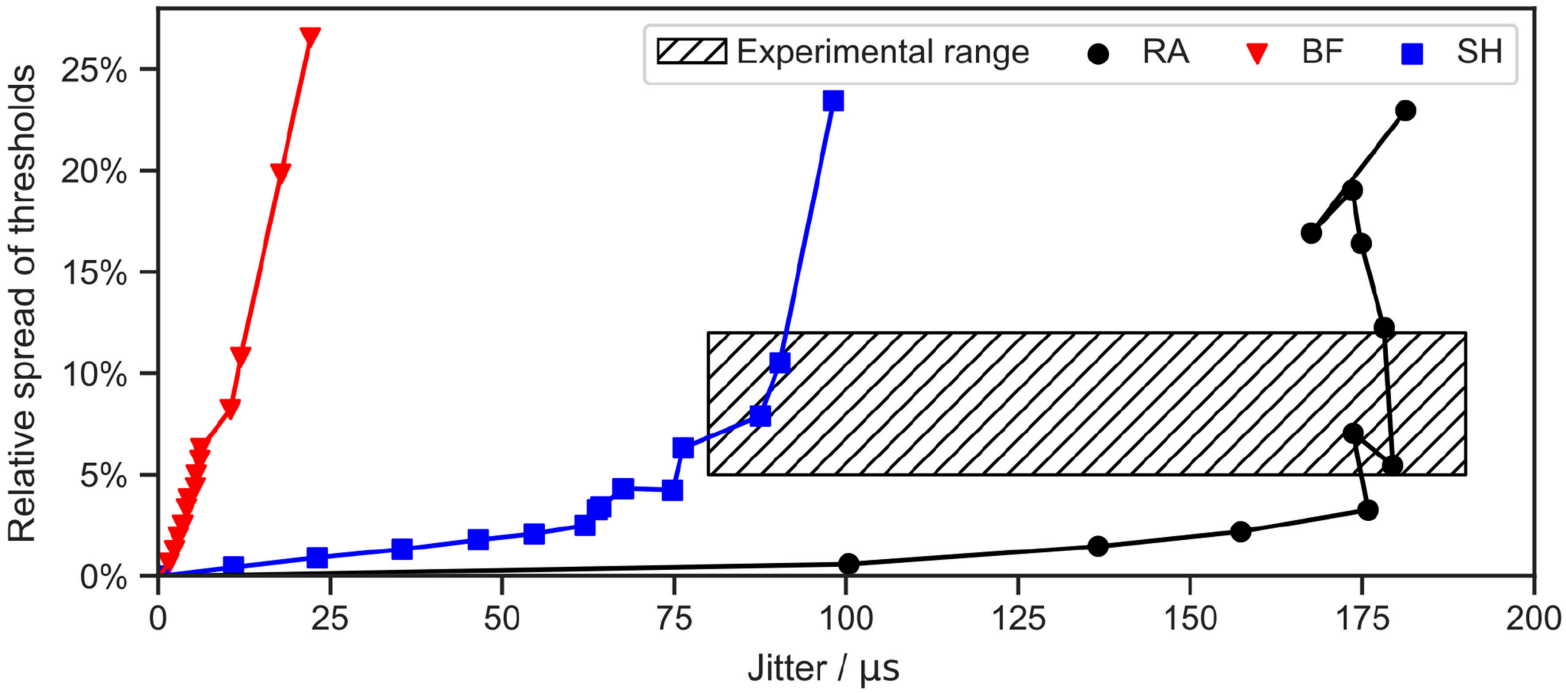
Stochasticity of ANF models with a Gaussian noise current term. Jitter and relative spread of threshold were measured for different values of *k*_*noise*_. A monophasic 50 μs cathodic current pulse was applied in each simulation. Threshold and latency were measured 100 and 500 times, respectively, for each data point. “RA,” “BF,” and “SH” denote the Rattay, Briaire-Frijns and Smit-Hanekom models, respectively. The experimental range was summarized from a series of animal experiments, including van den Honert and Stypulkowski (1984), Javel et al. (1987), Dynes (1996), Miller et al. (1999), and Cartee et al. (2000).

The stochasticity of ANFs can be described with two aspects: one is the jitter, defined as the standard deviation of repeated measurements of the latency; the other is the relative spread of the threshold *I*_*th*_, calculated as the standard deviation of the threshold measurements divided by the mean (van Gendt et al., 2016). In this section, the Gaussian noise current term proposed by Rattay et al. (2001b) was added to all three ANF models, as we wanted to investigate whether this simple and computationally efficient approach was sufficient to simulate the stochastic behavior within the range of experimental measurements. Monophasic 50 μs cathodic current pulses were used for simulations, and stochastic behaviors were recorded for various values of *k*_*noise*_, ranging from 0.1 to 2 times the initial value which was fitted in order to obtain a relative spread of about 5%. Threshold measurements for each *k*_*noise*_ value were repeated 500 times to calculate the relative spread. Jitters were obtained by measuring the latency 500 times for a stimulation with *I*_*th*_. Spontaneous APs, i.e., APs initiated at 0 A or before the onset of the stimulus, were excluded in both measurements. Results are illustrated in Figure 9.

For the selected range of *k*_*noise*_, the relative spread lay below 30 % for all models. Further increases in *k*_*noise*_ can result in larger spreads but also in a high probability for spontaneous APs. In comparison, results for the jitter were more varied. While the jitter could reach as far as 180 μs with RA, it was confined to 25 μs in the case of the BF model.

Javel et al. (1987) reported a relative spread of 12 % and 11 % in feline ANFs using biphasic stimuli with phase durations of 200 and 400 μs, respectively. Smaller values between 5% and 10% were found by Miller et al. (1999) and Dynes (1996), who excited feline ANFs using monophasic pulses with a phase duration of 100 and 40 μs. Experimentally observed jitters for a stimulation of feline ANFs with *I*_*th*_ ranged from 80 μs (Cartee et al., 2000) to 190 μs (van den Honert and Stypulkowski, 1984). Hence, the addition of Gaussian noise current to RA and SH with appropriate values for *k*_*noise*_ managed to produce both relative spread and jitter that fit the experimental range, as shown in Figure 9. However, the jitter generated by BF was too small even for high *k*_*noise*_ values.

### 3.7. Pulse-Train Responses and Adaptation

In this section, the spiking behavior of the ANF models was investigated for pulse-train stimulations. The Gaussian noise current term was again added to all models to account for the stochasticity. Biphasic current pulses with a phase duration of 20 μs and an amplitude of 1.5 *I*_*th*_ were used.

**Figure 10:**
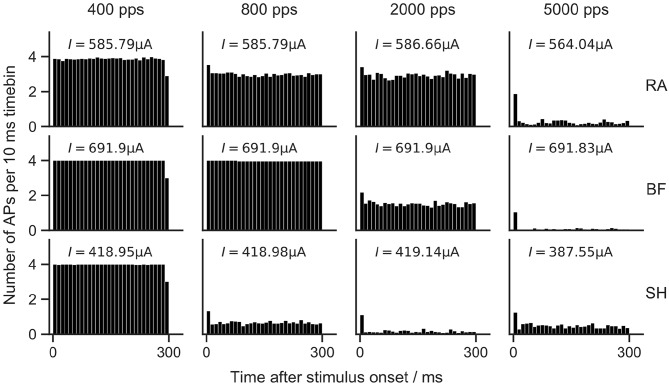
Poststimulus time histograms of ANF models to 300 ms pulse-train stimulation. RA, Rattay model; BF, Briaire-Frijns model; SH, Smit-Hanekom model. Biphasic (cathodic-first) current pulses with a phase duration of 20 μs and an amplitude of *I*_*th*_ were used for pulse-trains with four different pulse rates. Each stimulation was repeated 50 times. Vertical columns in PSTHs show the average number of APs in a 10 ms time bin.

The train of pulses lasted for 300 ms, and four different pulse rates were investigated. Each stimulation was repeated 50 times. Poststimulus time histograms (PSTHs) were used to depict the average number of APs in each 10 ms time bin in Figure 10.

In general, higher pulse rates led to reduced firing efficiency. With a rate of 400 pps, 100% firing efficiency was obtained in all models. For an increase to 800 pps, RA and SH predicted reduced firing rates. With a further increase to 2,000 pps, RA showed a similar spiking behavior as for 800 pps, while the spiking rate of BF was reduced by more than a factor of two, and SH responded almost solely to the first pulses of the pulse trains. When stimulated with 5,000 pps, small firing rates were measured with all models.

Adaptation of ANF spiking rate has been demonstrated in animal experiments. Zhang et al. (2007) measured adaptive responses to pulse trains with rates between 250 and 10,000 pps, and reported that the reduction in firing rates became larger as pulse rates increased. A similar tendency was observed by Litvak et al. (2001), who applied pulse-train stimuli with rates of 1,200 and 4,800 pps. Zhang et al. (2007) and Westerman and Smith (1984) concluded using feline and gerbil ANFs that adaptation was strongest during the first 10 ms of a pulse train, but still apparent after 100 ms. As none of the ANF models used in this study were explicitly developed to include adaptation, it is unsurprising that they showed no or little adaptation mostly limited to a reduction in firing efficiency following the first AP.

## 4. Discussion and Conclusion

In this study, we designed a computational framework to investigate some properties of biophysical multi-compartment models of the human ANF. We subsequently implemented three existing cable models in this framework, including RA (Rattay et al., 2001b), BF (Briaire and Frijns, 2005) and SH (Smit et al., 2010), and compared the outcomes with each other and with experimental measurements. This is the first study to perform a systematic comparison between different multi-compartment models of the human ANF, and will contribute to the future development of ANF models.

In comparison to experimental data, ANF models predicted drastically smaller ratios between ARP and RRP values as they revealed an overestimated ARP and an underestimated RRP. With axon models by Frijns et al. (1994) and Imennov and Rubinstein (2009), distinctly higher ratios of RRP to ARP have been predicted (detailed results not shown). A likely explanation for the more physiologically accurate refractoriness of axon models is the simplified morphology, particularly the lack of a soma. Moving the stimulus location for the human ANF models from dendrite to axon and therefore excluding the delay resulting from conduction across the soma region would have led to less steep refractory curves and more physiological ARP and RRP values. One exception may be the SH model, whose ARP was twice the magnitude of the other models. This large ARP is likely to be associated with the long AP duration exhibited by SH (approximately 1 ms, as shown in Figure 7), whereas the other two models presented a much shorter AP duration (approximately 1/3 ms). The long AP duration thus makes it impossible for the SH model to achieve the experimental ARP value of 300 μs to 500 μs. Moreover, computational studies demonstrated that the cathodic and anodic thresholds (and their ratio) varied, as the stimulus shifted in constant distance along the axis of a cell (Rattay, 1999), or even as it moved along a fiber with constant diameter (Rattay, 2008). Since the chronaxie is rather different between myelinated axons and the non-myelinated soma (Ranck, 1975; Rattay et al., 2012), moving the stimulation site also altered the strength-duration relationship of the neuron. As a consequence, model validation may only be sensible when the stimulation conditions are comparable in both the models and the experiments.

One major hindrance regarding human ANF modeling is that neither the precise morphology nor the ion channel kinetics of human neurons are completely characterized (O'Brien and Rubinstein, 2016). In general, the internode length increases rather proportional with axon diameter (Rushton, 1951). The SH model, in which a shorter internode was attached to a thicker central axon compared to the peripheral axon, is thus in conflict with this observation. The inclusion of a soma is crucial for a realistic description of the human ANF; this necessitates the addition of a dendrite, which further complicates the optimization of an already large set of parameters in biophysical ANF models. The soma (unmyelinated but surrounded by layers of “satellite cells,” as described in Rattay et al., 2001b) in human ANF models is highly capacitive and thus charge consuming, which imposes a huge barrier for the propagation of an AP. This leads to a large delay in propagation. Rattay et al. (2001b) mentioned that the somatic barrier became insurmountable for APs after only small variations of certain model parameters. This reveals the difficulty of balancing the capacity of the soma in order to predict a realistic somatic delay without erasing the AP. Even small changes in the stimulation pattern such as an increase of the IPI for a few microseconds can cause the loss of the second AP at the somatic region, which explains the very steep refractory curves as shown in Figure 8. Somas in feline ANF models are less critical for the propagation of APs as they are small and myelinated (Liberman and Oliver, 1984), which reduces the capacity and in turn the chance of losing an AP at the somatic region. A shorter presomatic delay was reported when the somatic diameter in the RA model was reduced from 30 μm to 20 μm, which was closer to average soma size of human spiral ganglion cell, and thus the temporal spiking behavior was altered when the soma diameter was changed (Potrusil et al., 2012). Furthermore, the conduction velocity was also influenced by the axon diameter. An increase in the respective diameter of peripheral and central axons in RA from 1 and 2 μm to 1.3 and 2.6 μm, which was closer to measurements from human specimen, decreased the conduction time by 21.4 % (Rattay et al., 2013).

In this study, the Gaussian noise current term in RA was also applied to the other two models to account for the stochastic nature of ion channels. Based on Equation (1), this noise current increases with the maximum sodium conductivity and the membrane surface area, implying that stochasticity is more pronounced in larger fibers and with higher sodium densities. However, the contrary has been revealed in experiments: the strength of stochasticity was found to decrease as the fiber diameter increased (Verveen, 1962), and the relative spread was later demonstrated to be inversely proportional to the square root of the total number of sodium channels (Rubinstein, 1995). As a consequence, the role of a single channel in the voltage fluctuation is less significant when compared to the total ionic conductance (Rubinstein, 1995; Badenhorst et al., 2016). Moreover, experiments showed that the ionic channel noise of ANF increased as the membrane potential deviated from the resting potential (Verveen and Derksen, 1968), but such voltage dependency was not included in the noise current term by Rattay et al. (2001b). A modified version of the conductance-based stochastic model, which included the inverse relationship and voltage dependency, has been proposed by Badenhorst et al. (2016). Here, the authors were particularly motivated to have their model reflect the actual *in vivo* behaviors. The single node model by Negm and Bruce (2014) and the axon model by Imennov and Rubinstein (2009) produced stochastic responses using a channel number tracking algorithm with channel transitions following a Markov jumping process. This approach was found to be the most accurate one to model channel noise (Mino et al., 2002). It is hence worth further investigating the applicability of these approaches in our framework.

None of the three models predicted pulse-train responses in a range comparable with experimental results, because they were not able to appropriately account for temporal effects of ANF, such as pulse-train integration or adaptation. Therefore, these models need to incorporate a mechanism capable of predicting such long-term effects, as these effects are likely to exert an significant impact on the perception of CI users (Clay and Brown, 2007). Currently, there is still no precise knowledge regarding the mechanisms of the adaptive behavior observed in ANFs. Nevertheless, two biophysical approaches for adaptation have been developed. Woo et al. (2009) modeled adaptation using a dynamic external potassium concentration ${\left[K^{+}\right]}_{e}$ at the nodes of Ranvier and applied it to a feline ANF model in Woo et al. (2010). The model was based on the findings on leeches that ${\left[K^{+}\right]}_{e}$ changes induced adaptation-like effects (Baylor and Nicholls, 1969). However, there is no experimental evidence that an ongoing stimulation of a nerve fiber can alter ${\left[K^{+}\right]}_{e}$ sufficiently, or that this is the case in mammal ANFs.

Negm and Bruce (2014) incorporated adaptation in a single node model by adding hyperpolarization-activated cation channels and low-threshold potassium channels, both of which have been identified in mammalian spiral ganglion neurons. These two types of ion channels had a much slower gating property and complemented the relatively fast dynamics of sodium and potassium currents. As this approach has not yet been applied to a multi-compartment ANF model, it remains unclear how the additional ion channels will affect the initiation and propagation of APs. A simple inclusion of these channels to an existing ANF model is not sufficient, as the spiking behavior of the model may be altered, and subsequently extensive parameter optimization is required. On the other hand, stochasticity and temporal behaviors of ANF have been efficiently implemented in phenomenological models. van Gendt et al. (2016) created a hybrid model that combined the biophysical and phenomenological approaches to efficiently predict responses to pulse-train stimuli. This model was also implemented in combination with a three-dimensional volume conduction model of the cochlea (van Gendt et al., 2016, 2017). Nonetheless, as phenomenological models do not include realistic biophysical details in their implementation, their predictions are often limited only to predefined stimuli.

## Data Availability Statement

The scripts and generated datasets for this study can be found at https://gitlab.lrz.de/tueibai-public/human-anf-models.git.

## Author Contributions

RB contributed to model simulation, data acquisition and analysis, and manuscript drafting. JE contributed to study design, data analysis, and manuscript revising. MO-L contributed to data analysis and manuscript revising. WH and SB contributed to study design and critical manuscript revising. The final manuscript has been approved by all authors.

### Conflict of Interest

The authors declare that the research was conducted in the absence of any commercial or financial relationships that could be construed as a potential conflict of interest.
