# Supplementary material for: Comparison of Multi-Compartment Cable Models of Human Auditory Nerve Fibers
Source: Front Neurosci. 2019 Nov 5;13:1173. doi: 10.3389/fnins.2019.01173 (PMC6848226; doi:10.3389/fnins.2019.01173)
Supplement: Supplementary file 1 [file Table_1.DOCX]

**Appendix Table I: Morphometric parameters of auditory nerve fibre models**

|  |  | **RA** | **BF** | **SH** |
| --- | --- | --- | --- | --- |
| **Quantities** | peripheral intenodes | 6 | 6 | 5 |
|  | central internodes | 20 | 30 | 100 |
|  | compartments per internode | 1 | 1 | 1 |
|  | presomatic region compartments | 3 | 10 | 3 |
|  | myelin layers peripheral internodes | 40 | - | 40 |
|  | myelin layers presomatic region | 0 | 4 | 0 |
|  | satellite cell layers soma | 3 | 4 | 3 |
|  | myelin layers central internodes | 80 | - | 54 |
| **Diameters (µm)** | peripheral axon | 1 | 3 | 1 |
|  | peripheral axon outer diameter internodes | 1.68 | (5) | 1.68 |
|  | central axon | 2 | 3 | 2.02 |
|  | central axon outer diameter internodes | 3.36 | (5) | 3.75 |
|  | soma | 30 | 10 | 27 |
|  | presomatic region | 1 | 2 | 1 |
| **Lengths (µm)** | peripheral terminal | 10 | 10 | 10 |
|  | peripheral nodes | 2.5 | 1 | 2.5 |
|  | cenral nodes | 2.5 | 1 | 1.061 |
|  | peripheral internode 1 | 430 | 250 | 210 |
|  | peripheral internode 2 | 430 | 250 | 440 |
|  | peripheral internode 3 | 430 | 250 | 350 |
|  | peripheral internode 4 | 430 | 150 | 430 |
|  | peripheral internode 5 | 430 | 100 | 360 |
|  | peripheral internode 6 | 360 | 50 | - |
|  | presomatic region | 100 | 100 | 100 |
|  | soma | 30 | 30 | 27 |
|  | postsomatic region | 5 | 0 | 5 |
|  | central internode 1 | 500 | 150 | 77.4 |
|  | central internode 2 | 500 | 200 | 77.4 |
|  | central internode 3 | 500 | 250 | 77.4 |
|  | central internode 4 | 500 | 300 | 77.4 |
|  | further central internodes | 500 | 350 | 77.4 |
| **Thickness (nm)** | thicknes myelin layers | 8.5 | - | 16 |

**Appendix Table II: Ionic channel kinetic parameters of auditory nerve fibre models**

|  |  |  |  | **RA** | **BF** | **SH axon** |
| --- | --- | --- | --- | --- | --- | --- |
| **temperature** |  | T | °C | (29) | 37 | 37 |
| **ionic concentrations** | sodium, intracellular | [Na+]_i | mol/m^3 | - | 10 | [Na+]_e / [Na+]_i = 7.210 |
|  | sodium, extracellular | [Na+]_e | mol/m^3 |  | 142 |  |
|  | potassium, intracellular | [K+]_i | mol/m^3 |  | 141 | K+]_e / [K+]_i = 0.036 |
|  | potassium, extracellular | [K+]_e | mol/m^3 |  | 4.2 |  |
|  | leak, intracellular | [Leak]_i | mol/m^3 |  | - | [Leak]_e / [Leak]_i = 0.0367 |
|  | leak, extracellular | [Leak]_e | mol/m^3 |  |  |  |
| **ion permeabilities** | nodal sodium permeability | P_Na | µm/s | - | 51.5 | - |
|  | nodal potassium permeability | P_K | µm/s |  | 2 |  |
| **maximum conductances unmyelinated terminal, nodes, pre- and postsomatic compartments (if present)** | sodium channel | g_Na | mS/cm^2 | 1200 | - | 656.7 |
|  | fast potassium channel | g_K bzw. g_K_f | mS/cm^2 | 360 |  | - |
|  | slow potassium channel | g_K_s | mS/cm^2 | - |  | 77.22 |
|  | leakage channel | g_L | mS/cm^2 | 3 | 72.8 | 90.54 |
| **maximum conductances soma** | sodium channel | g_Na | mS/cm^2 | 120 | - | 656.7 |
|  | potassium channel | g_K | mS/cm^2 | 36 |  | 77.22 |
|  | leakage channel | g_L | mS/cm^2 | 0.3 | G_L =1.13 in [nS] | 90.54 |
| **Nernst/Resting-potentials** | sodium | V_Na | mV | 115 | - | V_nerst - V_res |
|  | potassium | V_K | mV | -12 |  | V_nerst - V_res |
|  | leakage | V_L | mV | 10.6 | about 0 | V_nerst - V_res |
|  | membrane resting potential | V_res | mV | 65 | Goldman equation | -88.24 |
| **other constants** | capacitance per aria cell membrane (one layer) | c_m | µF/cm^2 | 1 | 2.801 | 2.8 |
|  | capacitance per aria myelin sheath | c_my | µF/cm^2 | 1 | 0 | 0.6 |
|  | membrane conductivity internodes | g_m | mS/cm^2 | 1 | 0 | - |
|  | membrane resistivity internodes | r_m | kΩ*cm^2 | - | - | 35.553 |
|  | myelin membrane resistance per aria | r_my | Ω*cm^2 | - | - | 0.7591 |
|  | intracellular (axoplasm) resistivity | rho_in | kΩ*cm | 0.05 | 0.07 | 0.025 |
|  | extracellular resistivity | rho_out | kΩ*cm | 0.3 | 0.3 | 0.3 |
| **ionic currents (per aria)** | sodium current | i_Na | A/cm^2 | g_Na*m^3*h*(V_m-V_Na) | Schwarz-Eikhof | 0.975*g_Na*m^3*h*(V_m-V_Na) |
|  | fast potassium current | i_K | A/cm^2 | g_K*n^4*(V_m-V_K) | Schwarz-Eikhof | - |
|  | slow potassium current | i_K | A/cm^2 | - | - | g_K*n^4*(V_m-V_K) |
|  | leakage current | i_L | A/cm^2 | g_l*(V-V_L) | G_l*(V-V_L) | g_l*(V-V_L) |
|  | persistent sodium current | i_Na_p | A/cm^2 | - | - | 0.025*g_Na*m_p^3*h*(V_m-V_Na) |
| **values to be calculated for every compartment** | membrane surface aria | A_m | cm^2 | see morphometric data | see morphometric data | see morphometric data |
|  | cross-section aria of axon | A_cross | cm^2 | see morphometric data | see morphometric data | see morphometric data |
|  | capacitance cell membrane nodes | C_m_node | µF | C_m = A_m*c_m | C_m = A_m*c_m [commend!] | C_m = A_m*c_m |
|  | capacitance membrane/myelin sheath internodes | C_m_internode | µF | C_m = A_m*c_m/(1+N) | 0 | C_m = A_m*[(1/c_m) + (N/c_my)]^-1 |
|  | conductance membrane internodes | G_m | mS | G_m = A_m*g_m/(1+N) | 0 | G_m = A_m*1/(N*r_my + r_m) |
|  | ion current | I_ion | mA | I_ion = A_m*i_ion | I_ion = A_m*i_ion | I_ion = A_m*i_ion |
|  | axoplasm resistance | R_a | kΩ | R_a = (len*rho_in)/A_cross | R_a = (len*rho_in)/A_cross | R_a = (len*rho_in)/A_cross |

**Appendix Table III: Equations for rate constants of auditory nerve fibre models**

|  |  | **RA** | | **BF** | | **SH axon** | |
| --- | --- | --- | --- | --- | --- | --- | --- |
|  |  | **value** | **formula** | **value** | **formula** | **value** | **formula** |
| alpha_m | A | 2.5 | (A-B*V_m)/(C*exp(A-B*V_m)-1) | 0.49 | A*(V_m-B)/(1-exp((B-V_m)/C)) | 4.42 | A*(B-C*V_m)/(D*(exp(B-C*V_m))-1) |
|  | B | 0.1 |  | 25.41 |  | 2.5 |  |
|  | C | 1 |  | 6.06 |  | 0.1 |  |
|  | D | - |  | - |  | 1 |  |
| alpha_m_p | A | - | - | - | - | 2.06 | A*(B-C*(V_m-20 mV))/(D*(exp(B-C*(V_m-20 mV)))-1) |
|  | B | - |  | - |  | 2.5 |  |
|  | C | - |  | - |  | 0.1 |  |
|  | D | - |  | - |  | 1 |  |
| alpha_n | A | 0.1 | (A-B*V_m)/(exp(C*(A-B*V_m))-1) | 0.02 | A*(V_m-B)/(1-exp((B-V_m)/C)) | - | - |
|  | B | 0.01 |  | 35.00 |  | - |  |
|  | C | 10 |  | 10.00 |  | - |  |
| alpha_n_s | A | - | - | - | - | 0.2 | A*(B-C*V_m)/(D*(exp(B-C*V_m)-1)) |
|  | B | - |  | - |  | 1.0 |  |
|  | C | - |  | - |  | 0.1 |  |
|  | D | - |  | - |  | 10 |  |
| alpha_h | A | 0.07 | A*exp(-V_m/B) | 0.09 | A*(B-V_m)/(1-exp((V_m-B)/C)) | 1.47 | A*B*exp(-V_m/C) |
|  | B | 20 |  | -27.74 |  | 0.07 |  |
|  | C | - |  | 9.06 |  | 20 |  |
| beta_m | A | 4 | A*exp(-V_m/B) | 1.04 | A*(B-V_m)/(1-exp((V_m-B)/C)) | 4.42 | A*B*exp(-V_m/C) |
|  | B | 18 |  | 21.00 |  | 4.0 |  |
|  | C | - |  | 9.41 |  | 18 |  |
| beta_m_p | A | - | - | - | - | 2.06 | A*B*exp(-(V_m-20 mV)/C) |
|  | B | - |  | - |  | 4.0 |  |
|  | C | - |  | - |  | 18 |  |
| beta_n | A | 0.125 | A*exp(-V_m/B) | 0.05 | A*(B-V_m)/(1-exp((V_m-B)/C)) | - | - |
|  | B | 80 |  | 10.00 |  | - |  |
|  | C | - |  | 10.00 |  | - |  |
| beta_n_s | A | - | - | - | - | 0.2 | A*B*exp(-V_m/C) |
|  | B | - |  | - |  | 0.125 |  |
|  | C | - |  | - |  | 80 |  |
| beta_h | A | 1 | A/(1+exp(B-C*V_m)) | 3.70 | A/(1+exp((B-V_m)/C)) | 1.47 | A/(1+exp(B-C*V_m)) |
|  | B | 3 |  | 56.00 |  | 3.0 |  |
|  | C | 0.1 |  | 12.50 |  | 0.1 |  |
